# Organizational cynicism and its relation to nurses' occupational burnout: Testing nurse managers' paradoxical leadership moderation effects

**DOI:** 10.3934/publichealth.2025017

**Published:** 2025-03-10

**Authors:** Wagih Mohamed Salama, Hazem Ahmed Khairy, Mohammad Gouda, Marwa Samir Sorour

**Affiliations:** 1 Department of Social Studies, College of Arts, King Faisal University, Saudi Arabia, Saudi Arabia; 2 Hotel Management Department, Faculty of Tourism and Hotels, University of Sadat City, Sadat City, Egypt; 3 Deanship of E-Learning and Information Technology, King Faisal University, Saudi Arabia; 4 Nursing Administration Department, Faculty of Nursing, Tanta University, Egypt

**Keywords:** nurses, occupational burnout, organizational cynicism, paradoxical leadership

## Abstract

**Background:**

One of the primary challenges that hinders organizational effectiveness and prosperity is organizational cynicism. Organizational cynicism is defined as a general or specific attitude of disappointment, insecurity, burnout, and mistrust towards individuals or groups. Paradoxical leadership, as applied by nurse managers, involves acknowledging and navigating the inherent tensions and contradictions within healthcare organizations, which enables managers to sustain balance in the workplace.

**Aim of the Study:**

This study aims to investigate the relation between organizational cynicism and a nurses' occupational burnout (testing nurse managers' paradoxical leadership moderation effects).

**Research design:**

A non-experimental cross-sectional prospective design was employed for this study.

**Subjects:**

A stratified random sample of 314 nurses participated in the study.

**Setting:**

The study was conducted at Main Tanta University Hospital, which is affiliated with the Ministry of Higher Education and Scientific Research.

**Method:**

Three tools were used for data collection: the Organizational Cynicism Scale, the Nurse Managers' Paradoxical Leadership Scale, and the Maslach Burnout Inventory (MBI).

**Results:**

A statistically significant positive correlation was found between organizational cynicism and occupational burnout. Additionally, a statistically significant negative correlation was observed between the nurse managers' paradoxical leadership and both organizational cynicism and occupational burnout.

**Recommendations:**

Healthcare leaders should take proactive measures to address organizational cynicism to mitigate a nurses' occupational burnout, which can contribute to the nursing shortage.

## 1. Introduction

The health segment is a unity of the most significant segments due to the critical function it theaters at the existing time [1]. Hospitals are exceedingly systematized systems that object to providing care to patients in dangerous situations [2]. Meanwhile, nurses waged in hospitals provide a chief slice of the health care for these patients, and they must be watchful enough to provide harmless services and exaltation patient protection [3]. Nurses are considered essential to healthcare organizations and nurse retention remains an encounter for the nursing administrators. Nurses who do not distinguish sufficient organizational provisions may exhibit undesirable manners toward their supervisors and organizations [4].

One of the topmost worries that hinder organizational effectiveness and success is organizational cynicism [5]. Organizational cynicism can be defined as universal or explicit manners of displeasure, diffidence, bleakness, irritation, suspicion of institutions or the public, and group cynicism [6]. The impression of organizational cynicism refers to employees' feelings of worthlessness in the workplace as well as job dissatisfaction [7]. In other words, organizational cynicism is an individual's negative attitude toward the organization in which they work [8].

Organizational cynicism is a negative attitude that is comprised of three main concepts. The first concept is cognitive (belief). It is the belief of the organization's lack of trustworthiness [8]. It entails the confidence that the organization's practices lack fairness, goodness, and honesty. The second concept is affective (emotional), which involves intense expressive reactions about the organization. The last concept is behavior, which refers to harmful propensities and chiefly embarrassing attitudes [9]. It consists of damaging and recurrently serious arrogance. Therefore, organizational cynicism is a learned response influenced by workplace practices to more occupational burnout [10].

Occupational burnout is a persistent work-related issue characterized by energy exhaustion, an increased spiritual distance, pessimism, cynicism, and a reduced professional efficiency [11]. Nurse burnout implicates the sensitive and physical tiredness that comes with the more accountabilities essential for nursing [12]. For nurses, burnout is the consequence of a high-stakes, demanding job that frequently exposes them to anthropological grief. Nurses realize death and heartbroken families every day and care with patients who are in physical and/or mental pain [13]. Furthermore, nurses work elongated shifts, often 12 or extra hours within one day. Wholly, those issues can lead to forceful burnout on their personal, expressive, and psychological exhaustion, self-loneliness, and a lack of sensation fulfilled or skilled in professional settings. Thus, bad surroundings such as not consuming real support or paradoxical leadership behavior within the workplace can make burnout even more [14].

Nurse Managers are persons accountable for superior-level administration for resounding the perception, objects, and ethics of the healthcare organization [15]. Nurse Managers use a variety of leadership styles to improve the quality of care [16]. They need to make a balance between the healthcare organization's demands and the nurses' needs [17]. The healthcare environment today is complex, competitive, and volatile. To overcome obstacles and successfully fulfill organizational requirements and nurses' demands, nurse managers must adopt various conflicting roles and apply paradoxical leadership styles in response to these competing demands and needs [18].

Paradoxical leadership is defined as a leading philosophy intended to equilibrate the contradictory and challenging demands within a healthcare organization. It is characterized as a “both/and” leading style and cognitive basis [19]. The “both-and” philosophy of paradoxical leadership refers to the behaviors of nurse managers being compatible with two paradoxes for nurse managers directing nurses: the paradox of hospital and the paradox of nurses, both at the same time and over time [20]. Paradoxical leadership includes the following five dimensions: treating nurses uniformly while allowing individualization; combining self-centeredness with other centeredness; enforcing work requirements while allowing flexibility; balancing decision-making with autonomy; and maintaining a balance between distance and closeness [21].

## 2. Significance of the study

This study contributes to the growing body of research on organizational behavior and nursing management by exploring the critical relationship between organizational cynicism and a nurses' occupational burnout [22]. Given the increasing prevalence of burnout in healthcare settings, especially among nursing staff, understanding the factors that contribute to this phenomenon is crucial to improve the staff's well-being and retention. The role of nurse managers in mitigating burnout is particularly important, as their leadership style can directly influence the work environment and organizational climate [23],[24]. By investigating the moderating role of paradoxical leadership, this study provides insights into how nurse managers can effectively manage conflicting demands and tensions within the healthcare environment to promote a healthier, more sustainable workplace. These findings are particularly relevant for healthcare administrators who seek evidence-based strategies to address burnout and improve organizational effectiveness.

## 3. Aim of the study

The existing study points to investigate the relation between organizational cynicism and a nurses' occupational burnout, focusing on testing the nurse managers' paradoxical leadership moderation effects.

## 4. Research questions and the conceptual model of the study

- What is the relation between organizational cynicism and the nurses' occupational burnout?
- What is the relation between the nurse managers' paradoxical leadership and organizational cynicism?
- What is the relation between the nurse managers' paradoxical leadership and the nurses' occupational burnout?
- Does the nurse managers' paradoxical leadership have a moderating role in the relation between organizational cynicism and a nurses' occupational burnout?

**Figure 1.**
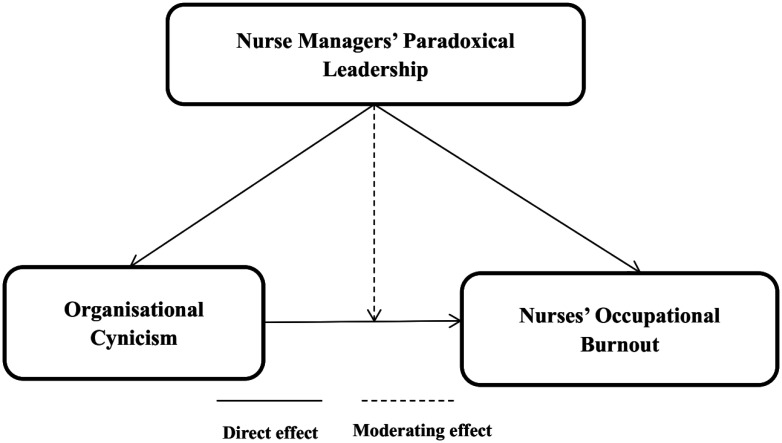
The conceptual model of the study.

Figure 1 exemplifies the conceptual framework of the current study:

## 5. Materials and methods

### 5.1. Research design

This study utilized a non-experimental, descriptive correlational, cross-sectional prospective design.

### 5.2. Setting

The study was conducted at Main Tanta University Hospital, a scientific research institution affiliated with the Ministry of Higher Education, and involved the Neurological department, Oncology department, Obstetric department, Cardiac department, Pediatric Hospital, and New Surgical Hospital. It is the biggest governmental hospital in El Gharbia, Delta region, Egypt. This hospital was chosen due to its comprehensive range of medical services and its affiliation with the Ministry of Higher Education, which ensures access to a diverse and representative sample of nurses across different specialties.

### 5.3. Subjects

A stratified random sample of 314 nurses (out of a total of 1699 nurses) was selected from various departments at Main Tanta University Hospital. The sample was stratified according to the number of nurses in each department to ensure that the sample was proportionally representative of the hospital's workforce. The study focused on full-time nurses who had spent at least six months in the same unit under their nurse manager.

The inclusion criteria for participation were as follows: registered nurses who have been employed at the hospital for at least one year, and nurses who work directly with patients in clinical settings. The exclusion criteria were as follows: nurses with less than one year of experience, and those working in administrative roles. For an equal selection probability, the sample size of nurses to include, considering the overall population of nurses, was calculated.

The sample size was determined using a power analysis conducted through the Epi-Info statistical program, which calculated the necessary sample size to achieve a 95% confidence level (*Z*-confidence level) and a margin of error of 0.05 (*d*-error proportion).

### 5.4. Data collection tools

#### 5.4.1. Part I: Profile data

This part was developed by the researchers to collect data such as age, marital status, level of education, department, and years of experience.

Data collection for this study was conducted using a pen and paper survey administered to the participants. The survey was distributed to nurses in their respective departments, with sufficient time allowed for completion. The survey included three primary scales:

#### 5.4.2. Tool I: Organizational cynicism scale

This tool was adapted from Dean et al. [25] and Brandes et al. [26]. It is used to assess the nurses' perception levels regarding organizational cynicism. It is encompassed of the 3 dimensions (23 items): cognitive (8 items), affective (8 items), and behavioral (7 items). Each item was rated on a 5-point Likert scale, ranging from 1 (strongly disagree) to 5 (strongly agree). The total score was calculated by summing the item scores, with higher scores indicating higher levels of organizational cynicism. The psychometric properties of this scale, tested in the native language (Arabic), have demonstrated good reliability (Cronbach's alpha = 0.929).

#### 5.4.3. Tool II: Nurse managers' paradoxical leadership scale

This tool was designed by Zhang et al., (2015) [27]. It was used to assess the nurse managers' paradoxical leadership as perceived by nurses and consisted of five dimensions (22 items): treating nurses uniformly while allowing individualization, combining self-centeredness with other-centeredness, maintaining decision control while allowing autonomy, enforcing work requirements while allowing flexibility, and maintaining both distance and closeness. The responses were rated on a 5-point Likert scale, from 1 (strongly disagree) to 5 (strongly agree). The scale score was derived by calculating the mean of all the item responses. This scale showed strong psychometric properties in its native language version (Cronbach's alpha = 0.928).

#### 5.4.4. Tool III: Maslach Burnout Inventory (MBI)

This tool was designed by Maslach et al. [28], which is an active means of verifying consistency and validity in spotting the incidence and evaluating the degree of burnout in service workers. It is a mental assessment tool comprised of 22 symptom items that affect occupational burnout. MBI measures 3 dimensions or subscales of burnout: Emotional Exhaustion (EE) (9 items), Depersonalization (DP) (5 items), and Personal Accomplishment (PA) (8 items). Each item was rated on a 5-point Likert scale, from 1 (strongly disagree) to 5 (strongly agree). The scores for each dimension were calculated by summing the responses within each factor. The Arabic version of the MBI has demonstrated excellent psychometric reliability (Cronbach's alpha = 0.916).

### 5.5. Data collection

The study was approved by the medical and nursing executives of Main Tanta University Hospital and all tools were translated into Arabic using the back-translation technique.

The data collection process was conducted over two months from June 2024 to July 2024 at Main Tanta University Hospital. A detailed, step-by-step procedure was followed to ensure accurate and ethical data collection while maintaining the participants' privacy.

The questionnaires were administered using a pen-and-paper format. The nurses were contacted during their scheduled morning shifts by research assistants who were not part of the nursing staff. At the beginning of each shift, the nurses were approached and invited to participate in the study. Participation was entirely voluntary, and informed oral consent was obtained before any questionnaire distributed. The nurses were given approximately 15–20 minutes during their shift to complete the questionnaire. They had the option to complete it either in a break room or in a quiet area, away from patient care activities, to ensure privacy. Upon completion, the nurses were asked to place their surveys in sealed envelopes to maintain confidentiality. Then, the completed surveys were collected by the research assistants or a designated trusted individual (e.g., a head nurse), who ensured that the surveys were handled discreetly. To ensure confidentiality, no identifying information was collected on the survey forms, and the completed surveys were kept in a secure location until they were entered into the database. Additionally, the researchers took all necessary precautions, including secure storage of the completed surveys in a locked filing cabinet, and data entry was performed using anonymized codes.

### 5.6. Data analysis

Descriptive statistics, including means and standard deviations, were calculated, and the missing data were handled via Multiple Imputation. Normality was assessed using statistical tests. Regression and moderation analyses were performed to examine the relationships between organizational cynicism, burnout, and paradoxical leadership. The data analysis was conducted using PLS-SEM with WarpPLS and SPSS. PLS-SEM is a robust technique suitable for models that do not meet the assumption of a multivariate normality and for datasets where normality issues might restrict traditional parametric techniques. As suggested by Birkinshaw et al. [29] and Acedo and Jones [30], PLS-SEM is a well-established method for research models that deal with non-normal data distributions. A Confirmatory Factor Analysis (CFA) was used to test the three-factor model's fit, with fit indices such as APC, ARS, AVIF, and GoF showing a good model fit. The results confirmed the robustness of the model and instruments.

The study addressed potential non-response bias by comparing early responses to late responses. A *t*-test was used to investigate any significant differences between these groups, and the results showed no statistically significant differences (*p* > 0.05), thus suggesting that a non-response bias was not an issue in the data. Further analysis of common method bias (CMB) was performed to determine whether common source variance could have affected the results. The analysis indicated that no single factor accounted for more than 50% of the variance in the data, confirming that common method bias did not significantly influence the study's findings.

## 6. Ethical considerations

Written permission was gained from the Tanta University Faculty of Nursing Scientific Research Ethical Committee and verbal permission from the altogether contributor nurses before gathering any data. Regarding the use of oral consent, we acknowledge that written consent is generally preferred. However, oral consent was obtained due to logistical and practical considerations in this study setting. Nurses were working in clinical environments, and the nature of the survey required a minimal disruption to their work. We ensured that the oral consent process was thoroughly explained to participants, who were made fully aware of the voluntary nature of their participation and their rights to confidentiality and anonymity. The decision to use oral consent was approved by the ethics committee and the hospital's administration. The object of the investigation was explained to the contributors, then the data was collected by the researchers. Namelessness and privacy of contributors' data were sure. Volunteer contributors in the research were secure to all contributors. They were informed about the ability to withdraw from the study at any period without an open-handed any reason.

## 7. Results

### 7.1. Participant's profile

The participant profile includes 314 individuals (see Table 1). In terms of age, most participants were between 35–44 years (43.6%), followed by those over 45 (26.1%). Regarding marital status, 33.4% were married and 29.0% were divorced. For education, 39.5% held a Bachelor of Nursing Science, and 35.4% had more than 20 years of experience. The majority worked in the New Surgical Hospital (47.5%), followed by the Cardiac Department (18.2%).

### 7.2. Descriptive statistics

**Figure 2.**
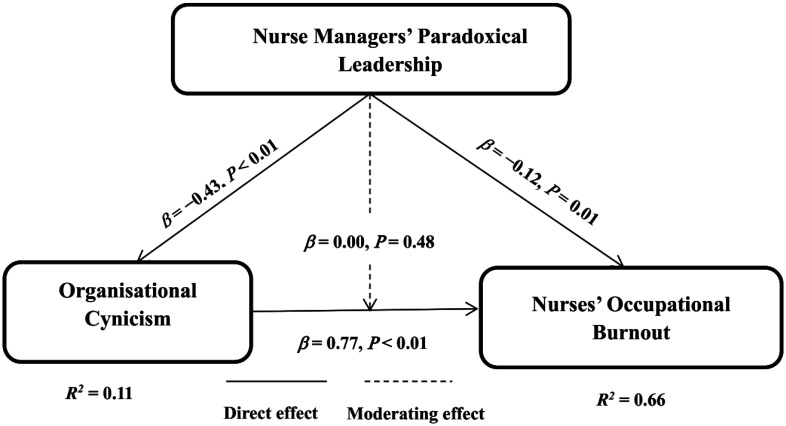
The final model of the study.

Table 2 displays the mean scores of organizational cynicism (OC), nurses' occupational burnout (OB), and nurse managers' paradoxical leadership (PL), (3.12 ± 0.764), (3.34 ± 0.657), and (2.99 ± 0.799). All variables had a moderate level since they fell between 2.34 and 3.66 (see Figure 2).

**Table 1.** Participant's profile (*n* = 314).

Items	Category	Frequency	Percent
Age	<25	46	14.6
	25–<35	49	15.6
	35–<45	137	43.6
	>45	82	26.1
Marital status	Single	49	15.6
	Married	105	33.4
	Divorced	91	29.0
	Widow	69	22.0
Educational level	Nursing school diploma	24	7.6
	Technical nursing degree	97	30.9
	Bachelor of nursing science	124	39.5
	Post-graduate nursing	69	22.0
Experience	6 months–<1 year	17	5.4
	1–<5 years	30	9.6
	5–<10 years	80	25.5
	10–<20 years	76	24.2
	>20 years	111	35.4
Department	Neurological department	22	7.0
	Oncology department	37	11.8
	Obstetric department	35	11.1
	Cardiac department	57	18.2
	Pediatric hospital	14	4.5
	New surgical hospital	149	47.5

**Table 2.** Descriptive statistics.

Construct	*Mean*	*SD*
Organizational Cynicism (OC)	**3.12**	**0.764**
1. Cognitive	3.17	0.866
2. Affective	3.05	0.796
3. Behavioral	3.11	0.911
Occupational Burnout (OB)	**3.34**	**0.657**
1. Emotional Exhaustion (EE)	3.39	0.731
2. Depersonalization (DP)	3.33	0.717
3. Personal Accomplishment (PA)	3.27	0.787
Paradoxical Leadership (PL)	**2.99**	**0.799**
1. Treating nurses uniformly while allowing individualization	2.96	0.971
2. Combining self-centeredness with other-centeredness	3.14	0.987
3. Maintaining decision control while allowing autonomy	2.97	0.982
4. Enforcing work requirements while allowing flexibility	2.91	1.121
5. Maintaining both distance and closeness	2.97	1.066

### 7.3. Measurement model

The three-factor model, including organizational cynicism (OC), nurses' occupational burnout (OB), and nurse managers' paradoxical leadership (PL), was tested using a confirmatory factor analysis. The model's fit was evaluated using Kock's [31] ten fit indices: APC “*p* < 0.05”, ARS “*p* < 0.05”, AARS “*p* < 0.05”, AVIF “acceptable if ≤5, ideally ≤3.3”, AFVIF “acceptable if ≤5, ideally ≤3.3”, GoF “small ≥ 0.1, medium ≥ 0.25, large ≥ 0.36”, SPR “acceptable if ≥ 0.7, ideally = 1”, RSCR “acceptable if ≥0.9, ideally = 1”, SSR “acceptable if ≥0.7”, and NLBCDR “acceptable if ≥0.7”. The proposed three-factor model provided data that was adequately fitted: “APC = 0.307, *p* < 0.001; ARS = 0.389, *p* < 0.001; AARS = 0.386, *p* < 0.001; AVIF = 1.162; AFVIF = 2.033; GoF = 0.449; SPR = 1.000; RSCR = 1.000; SSR = 1.000; and NLBCDR = 1.000”.

The research constructs high-reliability ratings (see Supplementary Table S1), with Cronbach's alpha and composite reliability ratings above the minimally acceptable level (CA > 0.70, CR > 0.70). In addition, the research constructs had statistically significant item loadings (item loading >0.60, *p* < 0.05). Additionally, the study confirmed convergent validity by AVE values (AVE > 0.50) for organizational cynicism, a nurses' occupational burnout, and the nurse managers' paradoxical leadership. The study's research model is considered free of common method bias, as the VIF for every latent variable is ≤3.3.

Moreover, by confirming that each construct's square root of the AVE is greater than the off-diagonal correlations, the discriminant validity of the constructs was confirmed (see Supplementary Table S2).

### 7.4. Path coefficients results

Figure 2 and Table 3 show that organizational cynicism (OC) positively affects a nurses' occupational burnout (OB) (*β* = 0.77, *β* < 0.01). This means that increased organizational cynicism leads to an increase in a nurses' occupational burnout. In addition, the nurse managers' paradoxical leadership (PL) negatively affects organizational cynicism (OC) (*β* = −0.43, *β* < 0.01) and a nurses' occupational burnout (OB) (*β* = −0.12, *β* = 0.02). This means that increased nurse managers' paradoxical leadership leads to a decrease in organizational cynicism and a nurses' occupational burnout. However, the nurse managers' paradoxical leadership does not moderate the relationship between organizational cynicism and a nurses' occupational burnout (*β* = 0.48).

**Table 3.** Path coefficients and *t*-values.

**Relationship**	**Direct effect (*β*)**	***Sig*.**	***t*-value**	**Decision**
*OC→OB*	0.77	p < 0.01	15.293	Supported
*PL→OC*	−0.34	p < 0.01	−6.298	Supported
*PL→OB*	−0.12	p = 0.02	−2.184	Supported
PL*OC→OB	0.00	p = 0.48	0.047	Not supported

Additionally, Figure 2 shows that the nurse managers' paradoxical leadership interpreted 11% of the variance in organizational cynicism (*R^2^* = 0.11). Moreover, the nurse managers' paradoxical leadership and organizational cynicism interpreted 66% of the variance in a nurses' occupational burnout (*R^2^* = 0.66).

## 8. Discussion

This study aimed to examine the relationship between organizational cynicism and occupational burnout, with a focus on paradoxical leadership as a moderator. The findings revealed that organizational cynicism positively correlates to a nurses' occupational burnout. These findings can be interpreted by the previous research of Simha et al. [32] and Alsubaie et al. [33], which claimed that organizational cynicism increases occupational burnout. Organizational cynicism and occupational burnout are interconnected issues that affect healthcare workers, especially in hospitals. Organizational cynicism involves a negative attitude towards an organization, management, or policies, while occupational burnout is a state of exhaustion caused by chronic workplace stress, characterized by exhaustion, cynicism, and reduced personal accomplishment. In other words, organizational cynicism and occupational burnout are closely linked. Organizational cynicism can contribute to occupational burnout in several ways: 1) Increased stress: A negative attitude towards the organization can lead to increased stress and job dissatisfaction; 2) Reduced job satisfaction: Cynicism can make it difficult for employees to find meaning or fulfillment in their work; and 3) Decreased motivation: A negative view of the organization can reduce employees' motivation to perform well.

Additionally, the findings revealed that the nurse managers' paradoxical leadership negatively correlates with both organizational cynicism and a nurses' occupational burnout. This finding can be interpreted by the findings of previous research by Pan [34] and Sulphey and Jasim [35], which argued that paradoxical leaders consistently treat subordinates without favoritism, thereby assigning a similar status and rights without favoritism. Chen and Yang [36] approached their study from a team-level cognitive process perspective, communicating, sharing views, and integrating opinions. They endowed subordinates with qualities such as adaptability, flexibility, and proactiveness, aiding their development and fostering innovation in complex team settings. In other words, paradoxical leadership, which is a leadership style that involves holding and embracing contradictory ideas or behaviors, can have a positive impact on healthcare organizations by reducing cynicism and occupational burnout among nurses. It can reduce negative attitudes, increase resilience, improve job satisfaction, and enhances organizational performance. However, it requires careful implementation and can be challenging to effectively execute, potentially leading to confusion, uncertainty, and increased stress among employees.

Lastly, this study revealed that paradoxical leadership does not moderate the relationship between organizational cynicism and burnout. Paradoxical leadership's effectiveness depends on the organization's context and organizational culture [37]. In organizations undergoing significant change or facing complex challenges, it may be more effective in mitigating cynicism and burnout. However, if the organization's culture is resistant to change or values conformity, paradoxical leadership may not be as effective [38]. In addition, the impact of paradoxical leadership may vary depending on individual differences, such as personality traits, values, and experiences. Some individuals may be more receptive to paradoxical leadership than others. In other words, although our initial hypothesis proposed that paradoxical leadership would moderate the relationship between organizational cynicism and burnout, the results did not support this moderating effect. Several factors could explain this finding. First, paradoxical leadership itself is a complex and multifaceted construct, and its effects may not be immediately apparent or may require a longer timeframe to manifest. It is also possible that other contextual variables, such as organizational culture or the specific challenges faced by the nursing staff at Main Tanta University Hospital, influenced the results. Additionally, the cross-sectional nature of the study limited our ability to capture the dynamic interactions between leadership styles and burnout over time.

In conclusion, while paradoxical leadership may not always directly moderate the relationship between organizational cynicism and burnout, it can still have positive effects on these outcomes, particularly in certain organizational contexts and when implemented effectively. Understanding these factors can help healthcare organizations leverage paradoxical leadership to improve an employee's well-being and organizational performance.

## 9. Conclusion

In the light of the present study, it could be concluded that the staff nurses' total mean scores of organizational cynicism, occupational burnout, and nurse managers' paradoxical leadership were at moderate mean scores. The existence of a moderation effect of the nurse managers' paradoxical leadership on the relationship between the independent variable (organizational cynicism) and the dependent variable (nurses' occupational burnout) was not supported. Moreover, the model suggested that organizational cynicism had a statistically positive effect on occupational burnout, while the nurse managers' paradoxical leadership had a statistically significant negative effect on both organizational cynicism and occupational burnout.

## 10. Practical implications (Recommendations)

On one hand, healthcare organizations can combat organizational cynicism and occupational burnout by implementing strategies that promote a positive work environment and reduce stress. These include improving communication, providing support, empowering employees, and promoting work-life balance. These measures can build trust, increase job satisfaction, and prevent burnout, thus ultimately leading to improved patient care and outcomes. On the other hand, to effectively use paradoxical leadership, healthcare organizations should train leaders, foster a supportive culture, encourage open communication, and provide clear expectations. Healthcare organizations can benefit from these strategies, thereby promoting a positive work environment for nurses.

## 11. Limitations and further research

This study's findings contribute to existing research on organizational cynicism, occupational burnout, and nurse managers' paradoxical leadership. The limitation of this study is the use of a single-hospital sample, which may limit the generalizability of the findings to other healthcare institutions or countries. Organizational and cultural differences could impact the outcomes, thus suggesting that the findings may not be easily replicable in different settings. Additionally, exploring the influence of cultural and organizational factors on these relationships would be valuable in broadening the applicability of the findings. Another limitation of this study is the reliance on self-reported questionnaires, which can introduce response biases such as social desirability.

Future research should explore the dynamic interactions between leadership styles and burnout by incorporating longitudinal designs to examine how paradoxical leadership may influence burnout and organizational cynicism over time. Future researches should aim to include a more diverse sample from various hospitals, regions, and countries to assess the consistency of these results across different healthcare contexts. Additionally, it should aim to benefit from incorporating mixed methods approaches (e.g., interviews or observational data) to reduce the likelihood of response bias. Using longitudinal designs would be beneficial in tracking these variables over time to better understand causal relationships and the lasting effects of leadership interventions on a nurse's well-being.

## Use of AI tools declaration

The authors declare they have not used artificial intelligence (AI) tools in the creation of this article.


