# Supplementary material for: Organizational cynicism and its relation to nurses' occupational burnout: Testing nurse managers' paradoxical leadership moderation effects
Source: AIMS Public Health. 2025 Mar 10;12(2):275–89. doi: 10.3934/publichealth.2025017 (PMC12277778; doi:10.3934/publichealth.2025017)
Supplement: Supplementary file 1 [file publichealth-12-02-017-s001.pdf]

Public Health

AIMS Public Health, 12(2): 275–289.
DOI: 10.3934/publichealth.2025017
Received: 26 September 2024
Revised: 10 December 2024
Accepted: 08 February 2025
Published: 10 March 2025

https://www.aimspress.com/journal/aimsph

Research article

Organizational cynicism and its relation to nurses' occupational burnout: Testing nurse managers' paradoxical leadership moderation effects

Wagih Mohamed Salama[1,]*, Hazem Ahmed Khairy[2,]*, Mohammad Gouda[3] and Marwa Samir Sorour[4]

[1] Department of Social Studies, College of Arts, King Faisal University, Saudi Arabia, Saudi Arabia
[2] Hotel Management Department, Faculty of Tourism and Hotels, University of Sadat City, Sadat City, Egypt
[3] Deanship of E-Learning and Information Technology, King Faisal University, Saudi Arabia
[4] Nursing Administration Department, Faculty of Nursing, Tanta University, Egypt

* **Correspondence:** Wagih Mohamed Salama: Email: welsayed@kfu.edu.sa; Tel: +9660546382082; Hazem Ahmed Khairy: Email: hazem.khaiery@fth.usc.edu.eg; Tel: +201002373889.

Supplementary

Table S1. Confirmatory factor analysis.

Construct	Composite reliability (CR)	Cronbach's alpha (CA)	Average variance extracted (AVE)	Variance inflation factors (VIF)
Organizational Cynicism (OC)	0.929	0.920	0.505	2.946
Occupational Burnout (OB)	0.916	0.903	0.511	2.937
Paradoxical Leadership (PL)	0.928	0.919	0.513	1.190

Table S2. Discriminant validity results - Fornell-Larcker Criterion.

Variables	OC	PL	OB
**Organizational Cynicism (OC)**	0.710	−0.332	0.605
**Paradoxical Leadership (PL)**	−0.332	0.716	−0.361
**Occupational Burnout (OB)**	0.605	−0.361	0.715

AIMS Press

© 2025 the Author(s), licensee AIMS Press. This is an open access article distributed under the terms of the Creative Commons Attribution License (https://creativecommons.org/licenses/by/4.0)
